# Exploring the views of young people with autism spectrum disorder (ASD) on how to improve medical consultations

**DOI:** 10.1192/bjo.2021.553

**Published:** 2021-06-18

**Authors:** Alexander Mayor

**Affiliations:** Leicestershire Partnership Trust, University of Exeter Medical School, Barnet, Enfield and Haringey Mental Health Trust, Camden and Islington NHS Foundation Trust

## Abstract

**Aims:**

The aim of this study was to explore experiences of consultations with non-specialist health professionals in a group of young people with ASD and their parents and what they considered would help to improve these interactions.

**Background:**

Research has shown that general awareness around autism is poor among the general medical community and this can result in poor communication during medical consultations.

**Method:**

An opportunistic group of 4 young people with ASD and 8 parents from a local support group in Exeter were interviewed in an informal environment about their experiences of healthcare consultations after seeking verbal consent.

**Result:**

Among the 8 parents interviewed the themes emerging were a deep lack of understanding and awareness among medical staff of the challenges faced by individuals with autism, the importance of the doctor-patient relationship to allow children to open up to healthcare professionals, and the need for all children to be respected as individuals.

Among the 4 young people (13 to 19 years) with ASD interviewed they identified significant anxiety around waiting for appointments and expressed a desire for a distractor to relieve stress, questions posed by clinicians were often vague and should be clearer, 3D models/mannequins could be useful to support understanding of anatomy and physiology, patience and a calm demeanour were vital with a quiet clinical environment to avoid distraction, time for mental preparation is important and efforts should be made to avoid delays or cancellation of appointments where possible.

An interactive website was generated in the light of feedback from the client group and their parents, aiming to educate clinicians regarding the challenges faced by this client group and provide a guide suggesting how to facilitate effective consultation through the use of simple techniques to promote engagement/reduce anxiety in the clinical environment.

**Conclusion:**

Simple changes to the approach to consultation with clients with ASD - a quiet consultation space, no delays and better communication - could reduce stress and promote positive interactions with a beneficial effect on healthcare delivery for this client group.

